# Looking Into Mona Lisa’s Smiling Eyes: Allusion to an Illusion

**DOI:** 10.3389/fnhum.2022.878288

**Published:** 2022-07-01

**Authors:** Daniele Zavagno, Rossana Actis-Grosso, Olga Daneyko

**Affiliations:** ^1^Department of Psychology, University of Milano-Bicocca, Milan, Italy; ^2^NeuroMI Milan Center for Neuroscience, University of Milano-Bicocca, Milan, Italy; ^3^BiPAC Centro Ricerche Patrimonio Storico e Culturale, University of Milano-Bicocca, Milan, Italy; ^4^Department of Psychology, Sociology and Politics, Sheffield Hallam University, Sheffield, United Kingdom

**Keywords:** Mona Lisa effect, staring portraits, picture perception, perspective robustness, facial expressions and emotion, gaze expression, gaze direction changes

## Abstract

We present results from two experiments aimed at studying the direction of *Mona Lisa*’s gaze and its affective expression. In experiment 1 we studied the effect of retinal image size on the perception of her gaze by manipulating observation distances of a high-quality print of the painting. Participants (*N* = 30) were asked to answer a simple question (is the person portrayed looking at you?) from six different distances ranging from 55 to 755 cm. One group of participants started evaluations from 55 cm; the other group did the opposite. Results show an effect of distance on the perception of Mona Lisa’s gaze as staring at the observer: from the furthest distances, the impression of a staring Mona Lisa is robust; from the nearest distances, such impression becomes ambiguous. Experiment 2 presents data concerning the direction of Mona Lisa’s gaze and whether this appears to be smiling, derived from an experiment aimed at studying the impression of gaze (direction and emotional content) in portraits (paintings and photographs). Only data concerning Mona Lisa are reported. Participants (*N* = 41) were randomly assigned to one of two groups: on a LCD screen, one group saw the entire head, and the other group saw only a section reproducing Mona Lisa’s eyes. Experimental sessions were two: in session 1 participants had to decide whether the image (whole-head or eyes only) was looking at them; in session 2 participants had to decide whether the head (or the eyes) was smiling. RTs from the two groups of participants were not statistically significant. Results for session 1 confirm experiment 1’s general findings. Results for session 2 clearly show that Mona Lisa is not only smiling with her face, but also with her eyes. Results are discussed in relation to the literature on Mona Lisa’s gaze and smile.

## Introduction

This article reports findings from two experiments concerning the direction of Mona Lisa’s gaze (experiments 1 and 2) and the expression of her eyes (experiment 2). Mona Lisa (or *Monna Lisa*), aka *La Gioconda*, is one of the world’s most iconic artworks and also one of the most studied not only by art historians but also by vision scientists. The titles *Mona Lisa* and *La Gioconda* are both derived from Giorgio Vasari (1511–1574, Italian painter, architect, and art historian), best known for his book *Lives of the Most Excellent Painters, Sculptors, and Architects*. Vasari identified the sitter in Leonardo Da Vinci’s masterpiece as Lisa Gherardini, wife of Francesco del Giocondo. Hence, when Cassiano dal Pozzo (1588–1657, Italian scholar and art collector) wrote *d’una tal Gioconda* referring to the masterpiece he saw during his visit to Fontainebleau in 1625, he was most likely making a play of words, given that in Italian “giocondo” means both “playful” and “he who brings joy”. In more than one sense, *La Gioconda* has indeed proven to be playful (Pedretti, 1956).

While in more recent times several art historians have challenged Vasari’s account (e.g., Pedretti, 1956; Zapperi, 2012), troubling themselves in trying to figure out the real identity of the woman portrayed, several vision scientists have, instead, dabbled with the elusive quality of Mona Lisa’s facial expression, as if it were a visual illusion. The portrait, in fact, is considered to have an enigmatic facial expression, sometimes smiling, sometimes not, but always with her gaze fixed on the observer, whatever her/his position in front of the painting. The goal of most studies has been, therefore, to uncover the factors underlying the mobility of Mona Lisa’s expression. Research has focused mostly on her gaze or on her smile. As stated above, this article also deals with the direction of Mona Lisa’s gaze and with her smile, the last, however, still in reference to her gaze, seeking for an answer to the question: is she smiling with her eyes? Hence, this article will look into Mona Lisa’s eyes by reporting results from two studies. The first study, was devoted entirely to the direction of Mona Lisa’s gaze; in the second study, instead, we considered both her gaze and the affective expression of her eyes. The studies we present here were conducted before the sars-cov-2 outbreak.

## Looking at Mona Lisa from Different Distances

By googling “Mona Lisa effect” one finds over 43,000,000 results related to the phenomenon of a portrait’s eyes (be it a drawing, a painting, or a photograph) that appear to stare at those who are observing the picture, whatever their position and despite their moving about while looking at the picture. However, recently Horstmann and Loth (2019) have produced empirical evidence showing that the gaze of Mona Lisa is directed approximately at 15.4° to the right side of the observer. In other words, she should appear to be not looking at us. Nevertheless, not only the vulgate, but also several vision scientists believe, or at least take for granted, that Mona Lisa is staring at us (e.g., Rogers et al., 2003; Todorovic, 2006; Al Moubayed et al., 2012; Boyarskaya et al., 2015). In fact, several years ago, in a small workshop on the psychology of art, we also argued that Mona Lisa is not actually looking at the observer, claiming that her gaze is oriented to the right of the viewer’s head. To our astonishment, most of our peers were in total disagreement. We, therefore, started to review the issue by considering how people get to know about this masterpiece.

There are obviously several possibilities, but the most common is by seeing a reproduction in a book, a magazine, on the web, or by going to the Louvre. However, in the first three cases, reproductions are most likely heavily scaled in size, whilst in the last case, because of important security measures, Mona Lisa would be seen from an average distance of about 3–3.5 m. Considering that the painting measures 77 × 53 cm, the average retinal sizes of the entire painting would not go much beyond 14 × 10 deg during a visit to the Louvre, which is even smaller than the retinal sizes one may have by looking at a digital reproduction of the painting on a laptop. We, therefore, considered retinal size as the main factor that may impact the impression whether Mona Lisa is staring at the observer or not. To test this hypothesis, we decided to work with a more “ecological” setup, similar to one of the setups employed by Soranzo and Newberry (2015) to study *La bella principessa*, in which angular sizes were manipulated by the distance of observation of artworks.

### Participants

Thirty people (16 female) with an age range between 20 and 60 years (*M* = 20.03, SD = 8.27), all working or studying at the University of Milano-Bicocca, participated in the experiment. Participants were randomly assigned to one of two groups dubbed “near” and “far”, labels which refer to a participant’s first experimental trial. All participants had a normal or corrected-to-normal vision. None of the participants were aware of the purpose of the experiment, but some may have been aware of the so-called “Mona Lisa effect” because of prior familiarity with the portrait. However, such responses were not collected. All participants completed an informed consent with an overview of the experimental procedure. The experiment was conducted in accordance with the Declaration of Helsinki (World Medical Association, 1997).

### Material

**Figure 1:**
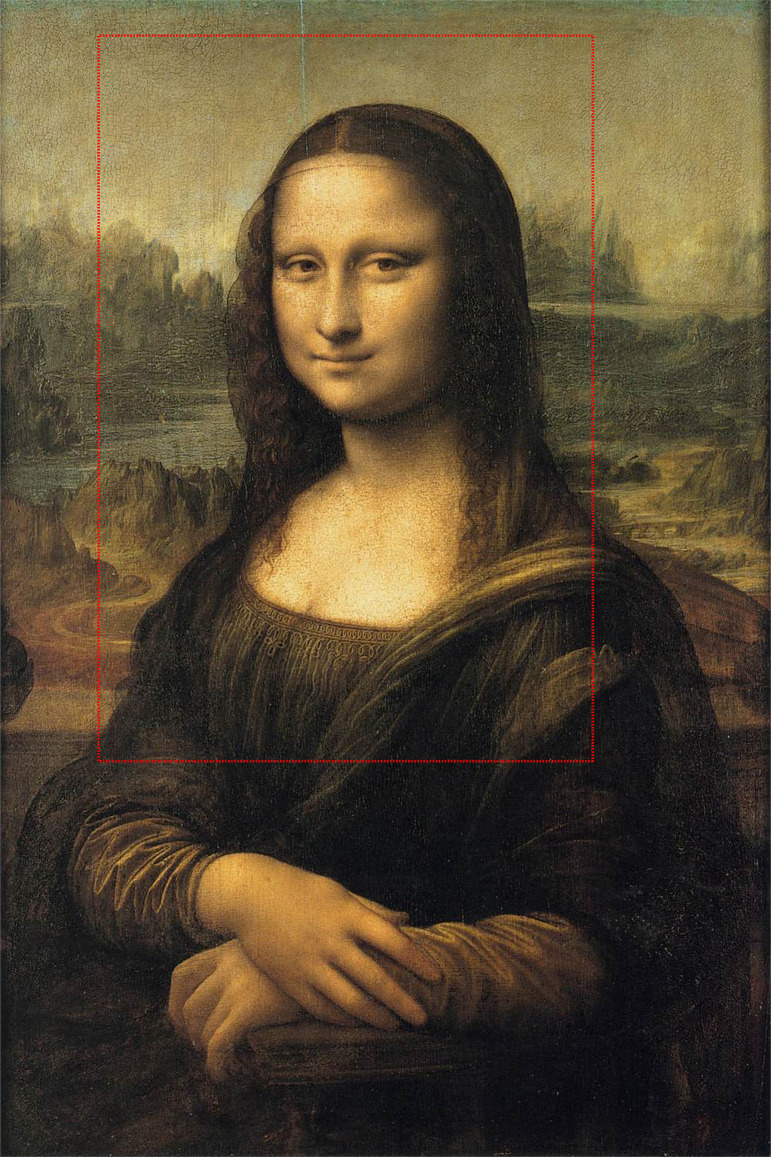
Leonardo Da Vinci (1452–1519), *Mona Lisa* (1503–5 ca, Musée du Louvre). The red rectangle delimits the area that was employed as stimulus.

The stimulus employed was a true-size high-definition inkjet print of Mona Lisa that included part of the background, torso, and hands (Figure 1). The print measured 48.3 × 33 cm and it was fixed on a rigid panel placed on a tripod.

### Experimental Design and Procedure

We employed a mixed design. The within factor was the viewing *distance* of the stimulus, which had six levels: 55 cm (47.41 × 33.39 deg), 110 cm (24.76 × 17.06 deg), 220 cm (12.52 × 8.57 deg), 420 cm (6.58 × 4.49), 520 cm (5.31 × 3.63 deg), and 755 cm (3.66 × 2.50 deg). The between factor was the *first distance* of observation, which was set at either 55 or 755 cm. The task was simply to respond to the following question: *Is the person portrayed looking at you?* Answers were dichotomous, being simply *yes* or *no*. Hence participants either started the experiment by looking at the stimulus from 55 cm and moving backward to look at it from each of the subsequent further distances, or, *vice versa*, by looking at the stimulus from 755 cm and moving forward to each of the next closest distances. The purpose of the within variable *distance* was to test for eventual changes in participants’ responses due to distance; the purpose of the between variable *first distance* was to control for an eventual carryover effect related to participants’ answers from the first two positions of observation.

The experiment took place in a long corridor of the Department of Psychology of the University of Milano-Bicocca. A text was read to the participant explaining that the task was to look at an image from different distances and to answer the question that would have been posed for each distance of observation with a simple “yes” or “no”. It was stressed that there were no right or wrong answers. The participant was then brought to the starting position of his/her group while instructed to look down at the floor. After answering the question from the first position, the participant was asked to stand up and to turn around so that their back was facing the stimulus. Hence, the chair was moved to the next position, which, based on the participant’s group, was either further away or closer to the stimulus. The participant was then guided to the next position. Positions were marked on the floor so that the chair would be positioned always in the exact same positions. There were no time constraints for answering the experimental question. The purpose of the study was explained to each participant when all trials were carried out. On an average, the entire procedure lasted from 20 to 30 min.

### Results and Discussion

**Figure 2:**
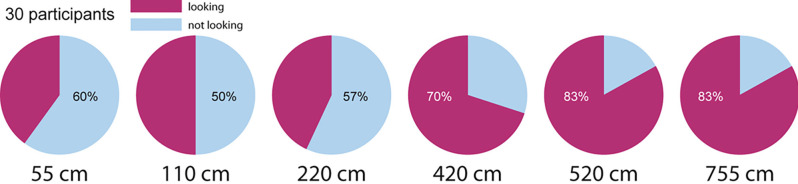
Percentages of yes-no replies from each distance to the question posed in experiment 1.

Figure 2 shows percentages of “yes” and “no” responses to the question “is the person portrayed looking at you?”: the first row shows overall results for the six distances, the second and third rows show data split by group.

Answers were analyzed with SPSS using a GEE binary logistic model for repeated measures, with *staring* (yes, no) as a categorical dependent variable, and *distance* and *first distance* as within and between independent factors, respectively. Wald χ^2^ statistics returned a significant effect on the model for *distance* (Χ^2^_(5,180)_ = 29.241, *p* < 0.001, *w* = 0.441, but neither for *first distance* (*p* > 0.4) nor the interaction first distance^*^distance (*p* > 0.3).

As one can see from Figure 2, there is a clear-cut difference between the three nearest distances (55–220 cm) and the three most remote distances (420–755 cm). Pairwise comparisons between the marginal means of the factor *distance*, conducted within the GEE model, confirmed such differences: estimated marginal means for distances 55, 110, and 220 are not statistically distinguishable (*p* > 0.07), but all three are significantly different from the estimated marginal means for distances 420, 520, and 755 cm (*p* < 0.05). The estimated marginal mean for distance of 420 cm is statistically different from the mean for 755 cm (*p* < 0.05), but not from that of distance 520 cm (*p* > 0.1). Moreover, Χ^2^ tests conducted to verify differences between *yes* and *no* responses confirm that distances 55–220 cm are ambiguous (*p* > 0.2), whilst distances 420–755 cm show a statistical difference in participants answers, in favor to the response *yes* (Mona Lisa is looking): 420 cm Χ^2^_(1,30)_ = 4.8, *p* < 0.028; 520 cm, *w* = 0.444; 520 cm Χ^2^_(1,30)_ = 13.33, *p* < 0.001, *w* = 0.666; 755 cm Χ^2^_(1,30)_ = 13.33, *p* < 0.001, *w* = 0.666.

When looking at the portrait from the two greatest distances (520–755 cm), the impression reported by most participants from both groups is that Mona Lisa is staring at them. This impression decreases significantly from position 220 cm, suggesting that Mona Lisa’s gaze appears intrinsically ambiguous from closer distances, while she appears to be mostly staring back at the observer when looked at from greater distances. A possible explanation for the ambiguity of Mona Lisa’s gaze may be related to the position of her head with respect to her gaze that, as measured by Horstmann and Loth (2019) is diverted to the right of the observer, while her head is slightly turned to the left, showing more of its right side.

## Eyes Smiling, but Not at Us

Results from experiment 1 support our hypothesis, according to which the impression of the direction of Mona Lisa’s gaze may vary depending on the retinal size of the image. However, we also hypothesized that bigger retinal images of the picture would determine the impression that Mona Lisa is not looking at the observer.

Here we report data concerning Mona Lisa’s gaze derived from another experiment in which we studied both the direction and the smiling impression of gazes in both painted and photographic portraits. In this study, we did not manipulate the retinal size of a reproduction of *La Gioconda*, which was viewed from a constant distance; instead, we manipulated the portion of Mona Lisa’s head that was visible: a full head, or only a section with her eyes. Such manipulation allowed us to study the role of head position in understanding the direction of Mona Lisa’s gaze.

The general goal of the experiment, from which we extrapolated the data pertaining to Mona Lisa, was to study the role of gaze direction and expression in pictorial artifacts, factors that are known to impact mental models about a person’s state (Marino et al., 2015), and, therefore, may contribute to the interpretation and aesthetic appraisal of portraits.

With regards to the ambiguous impressions generated by Mona Lisa’s portrait, along with the direction of her gaze, also her smile is one of the aspects that has attracted the attention of several researchers. Kontsevich and Tyler (2004, p. 1493) report that Mona Lisa “is the best-known example of an expression at the ambiguity point between a happy and a sad dimension”. It is possible that different interpretations of Mona Lisa’s affective state are related to the effect determined by her lips, which appear to be more “smiling” when not directly looked at, that is when the area of interest is dominated by low spatial frequencies (Livingstone, 2000). When looking at Mona Lisa’s eyes, the projection of her lips is eccentric with respect to the center of one’s gaze where acuity is greater because of higher spatial frequencies. According to Livingstone, the elusive appearance of Mona Lisa’s affective expression is therefore related to her smile, and to Leonardo’s sfumato technique. Hence, the impression of Mona Lisa also smiling with her eyes may depend on the lips smiling more when not directly looked at.

To the best of our knowledge, Kontsevich and Tyler (2004) were the first to investigate whether Mona Lisa is also smiling with her eyes. They concluded that the impression one may have of Mona Lisa smiling also with her eyes is due to the mouth region: the eyes appear to be smiling only because her lips are smiling. But if we put together Livingstone’s account with the conclusion drawn by Kontsevich and Tyler, we get a rather interesting contradiction. If one stares at Mona Lisa’s lips, her smile dims; instead, if one looks at her eyes the smile on her lips increases, making her eyes also appear to be smiling. This means that if one looks at Mona Lisa’s lips, the eyes should not appear to be smiling, as the smile “fades”, according to Livingstone. Hence, if the eyes are not smiling by themselves, then the impression should be of a sad Mona Lisa when looking at her lips.

That the mouth influences the affective expression of a face, thus also modulating the expressiveness of the eyes, should not come as a surprise: faces are rather complex and dynamic gestalts, in which changes in a core feature, such as the mouth or the eyes, will affect the “whole” and also its “parts”. This said, the questions here are different: regardless of other facial features, is Mona Lisa’s gaze fixed on the observer? Is it smiling?

### Participants

Forty-one students (seven males) of the Psychology Department of Milano-Bicocca, age range 19–28 (*M* = 23; SD = 3.1), took part in the experiment. All had a normal or corrected-to-normal vision. Participants were randomly assigned to one of two groups dubbed *whole-head* and *eyes-only*, labels that denoted the type of stimuli there were shown. None of the participants were aware of the purpose of the experiment, which was explained to them in detail at the end of their experimental session. Prior to taking part in the experiment, all participants completed an informed consent with an overview of the experimental procedure. At the end of the second experimental session, the purpose of the experiment was disclosed. The experiment was conducted in accordance with the Declaration of Helsinki (World Medical Association, 1997).

### Material

**Figure 3:**
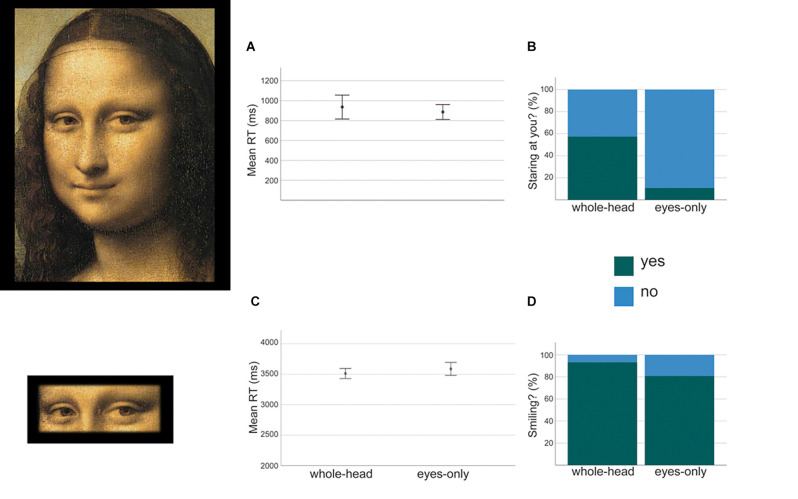
Example of the stimuli employed and the results for Mona Lisa from expriment 2. Panels **(A,B)** display mean RTs and percentage of yes-no answers referring to session 1 (staring). Panels **(C,D)** display mean RTs and percentage of yes-no answers referring to session 2 (smiling).

Stimuli for the group *whole-head* were derived from 24 portraits, 12 paintings (six male and six female portraits, among which there was, of course, *Mona Lisa*), and 12 black-and-white photographs of classic Hollywood movie stars (six male and six female). Stimuli were chosen based on a pilot study whose only purpose was to select the images to be employed in the actual study, from which the data pertaining to *Mona Lisa* has been extrapolated. For such a pilot study 10 participants were shown 20 painted and 20 photographic portraits and were asked to evaluate whether the portraits were looking at them. Portraits were selected based on two factors: clearly staring at the participant, or ambiguous (such as Mona Lisa). The heads of the selected portraits were then normalized so that facial features from all images were comparable in size, scaled down to fit a rectangle that measured 25.44 × 18.66 cm (27 × 20.4 deg) (h^*^b; Figure 3).

Stimuli for the group *eyes-only* were derived from the selected *whole-head* stimuli, but they displayed only the section of the face containing the eyes, which fitted a rectangle that measured 4.48 × 11.43 cm (5.12 × 12.8 deg; Figure 3).

All stimuli were seen from a distance of 50 cm against a black background on an “iMAC G5–17” with screen resolution 1,440 × 900 px.

### Experimental Design and Procedure

We employed a mixed design; however, in consideration of the fact that we are only interested in the results for Mona Lisa, only a between factor will be considered in this section, that is *group:* (1) participants who saw only *whole-head* stimuli; (2) participants who saw only *eyes-only*.

The experiment was divided into two sessions with a 5-min interval between them. Tasks were identical across groups. In session 1 the task was to decide whether the face (or the eyes for group *eyes-only*) was staring back at the participant by pressing the key labeled “yes” or “no” on a keyboard. The response limit in session 1 was set to 2 s; if the participant did not answer the trial was recorded as void and a new stimulus appeared. Stimuli within each set for session 1 were presented eight times in random order. In session 2 the task was to decide whether the face (or the eyes for group eyes-only) was smiling by pressing the same labeled keys. The response limit in session 2 was set to 5 s. Stimuli for session 2 were presented three times in random order. The average time for completing the experiment was approximately 50 min including the interval between sessions, initial debriefing, and final explanations.

### Results and Discussion

Response times (RT) were also collected along with the dichotomous responses to the questions: is the face (or the eyes) staring at you? (session 1); is the face (or the eyes) smiling? (session 2). Data from the two sessions concerning Mona Lisa are analyzed separately. We hypothesized that participants who saw the whole head would find it easier to decide whether Mona Lisa was looking at them or smiling with respect to those who saw only a section with her eyes.

*Session 1: staring*. Void trials and those in which RT < 100 were excluded from the datasets (five responses in total were excluded for *whole-head* and three *for* eyes-only). Mean RTs were calculated for participants from both groups and compared through a *t*-test for independent samples, which did not yield a significant difference between groups: Mean *whole-head* = 936 ms; M *eyes-only* = 887 ms; *t*_(39)_ = 0.739, *p* = 0.4, *d* = 0.2 (Figure 3A). Responses to session’s 1 question (staring) were analyzed with SPSS by means of a GEE binary logistic model for repeated measures in which *participant* served as a subject variable, *staring* as the dependent variable, and the *group* as between factor. The factor *group* (*whole head* vs. eyes only) determined a significant effect: Χ^2^_(1,320)_ = 23.867, *p* < 0.001, *w* = 0.273. Figure 3B show percentages of “yes” and “no” responses for the two groups. Eyes appear to be not staring when only the eyes were visible; instead, there is no statistical difference between yes and no responses when the whole head was visible.

*Session 2: smiling*. All RTs were considered as they were all greater than 2,000 ms. Mean RTs were calculated for participants from both groups and compared by means of a *t*-test for independent samples. As in session 1, RTs from the two groups are not statistically distinguishable: Mean *whole-head* = 3,511 ms; Mean *eyes-only* = 3,587 ms ; *t*_(39)_ = 2.247, *p* = 0.2, *d* = -0.3 (Figure 3C). Responses to session’s 2 question (smiling) were analyzed with SPSS through a GEE binary logistic model for repeated measures in which *participant* served as the subject variable, *smiling* as the dependent variable, and *group* as between factor. The factor *group* (*whole head* vs. eyes only) did not affect the model: Χ^2^_(1,123)_ = 2.664, *p* = 0.1, *w* = 0.147. Figure 3D shows percentages of “yes” and “no” responses for the two groups. The eyes appear to be smiling in both sets of stimuli.

The first consideration to make is that contrary to our expectations, response latencies were not significantly different between the two groups for both session tasks. We expected, in fact, both tasks to be relatively harder for group *eyes-only*, which would have reflected in significantly greater latencies.

Results from session 1 show that when only Mona Lisa’s eyes are visible the impression is that they are not staring at the observer, while when the whole head is visible results are ambiguous, with approximately only half of the participants seeing her *not staring* back at them (Figure 3B). These results suggest a major role of the position of the head in the perceived direction of Mona Lisa’s gaze, as suggested also by research on gaze direction and face eccentricity (Maruyama and Endo, 1983; Todorovic, 2009). Such a factor, however, is not able to override completely the impression that her gaze is not directed towards the observer. Hence, results confirm the findings for the closest positions in experiment 1, that is when retinal images of Mona Lisa’s face (and therefore of her eyes) are relatively big, and in particular when observers move closer to the portrait.

**Figure 4:**
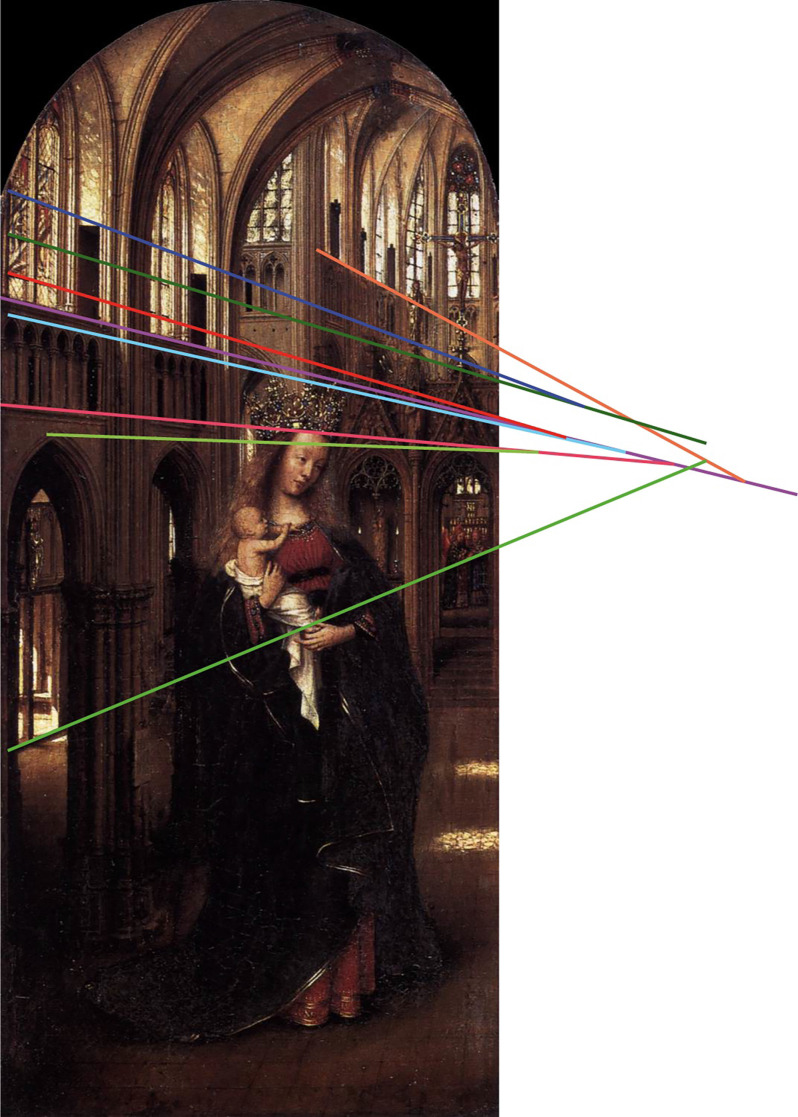
Jan van Eyck, *Madonna in the Church* (1425 ca., Staatliche Museen, Berlin). This modified image with superimposed straight lines shows that at the time the artist had a rather intuitive concept of what linear perspective is about: "vanishing points" appear to be many, but moving sideways with respect to the image does not determine the impression of spatial distortions. In other words, the layout of the image is “robust”.

Results from session 2 show that Mona Lisa’s eyes appear to be smiling by themselves (Figure 3D); in other words, though the smile on her lips certainly adds to the expressiveness of her eyes, these alone are, however, already intrinsically smiling. Our results do not support the claim by Kontsevich and Tyler (2004), according to which her eyes appear smiling only because of the smile on her lips. Our manipulation of the image portraying Mona Lisa was different from the aforementioned study (and also from the manipulation by Livingstone, 2000): we did not manipulate the spatial frequencies of the image, nor did we apply visual noise to it. Instead, we isolated the eyes from the rest of the image, whilst Kontsevich and Tyler selectively applied random noise to the image to determine “sad” or “happy” outcomes. Their Figure 4 (p. 1496) illustrates the problem related to such manipulations: while the noise applied to the lower part of the head alters dramatically the expression of the mouth, showing a frown in their panel A and an unnatural extended smile in their panel B, the noise applied to the upper part of the image does not alter in a significant way the expression of the eyes in their panels C and D. The conclusion they draw, that the smile affects the expression of the eyes, is therefore correct, but it tells nothing about the expressiveness of the eyes. By showing the eyes in isolation (Figure 3) we got rid of any effect by other facial features on their affective appearance, in particular, the effect due to the mouth: what our participants mostly saw were still smiling eyes.

## General Discussion

We offered an overview of the research we devoted to studying Mona Lisa’s gaze (specifically or indirectly), because we believe that, along with her smile, also her eyes play a central role in the aura of mystery that accompanies the perception of the painting.

Let us first consider findings relative to the expression of Mona Lisa’s eyes. In the second study along with gaze direction we also addressed the issue of whether eyes may appear to be smiling, even when only a pair of eyes are visible. At the time the experiment was conducted we were not living under Covid-19 restrictions so we could not anticipate how interesting the issue would have become of understanding another’s affective state just by looking at their eyes, in particular when the other person is wearing a mask covering mouth and nose. Considering only Mona Lisa’s case, the answer to the question of whether her eyes are smiling is positive: Mona Lisa’s eyes do carry a somewhat cheerful expression by themselves. This, however, does not mean that the expression depicted on Mona Lisa’s face cannot change into sadness if the mouth were to be modified, as demonstrated by Kontsevich and Tyler (2004). However, given that faces are indeed complex Gestalts, it might be interesting to see whether profound modifications to the expression of Mona Lisa’s eyes can also affect the overall affective interpretation of her facial expression, just as the changes in the expression of her mouth affected the expression of her eyes. However, all in all such manipulations would only tell us something about the perception of affective states, but nothing important about Da Vinci’s painting.

With regards to the issue of where Mona Lisa’s gaze is directed, our general hypothesis was that Mona Lisa is not staring at us, as the geometry explained by Horstmann and Loth (2019) informs us. However, given that most people believe that Mona Lisa is staring at them, we considered a more specific hypothesis related to retinal image sizes: we hypothesized that with relatively big retinal images of Mona Lisa’s head, the impression would be that her gaze is not directed at the observer, while with relatively small retinal images the impression would be the opposite (experiment 1). We clearly found the second case to be true. This finding appears to be opposite to the findings reported by Horstmann and Loth. One might think that the issue is related to how retinal sizes were manipulated: Horstmann and Loth manipulated retinal size without manipulating distance by zooming in and out of the portrait; we instead manipulated retinal size by manipulating viewing distances. However, such differences in manipulations should not determine different results given that even when the portrait’s angular size is reduced by increased viewing distance, the focus of attention is on the portrait, and it is unlikely that other visual information from a larger field of view would interfere with one’s impression of the direction of Mona Lisa’s gaze. We are left with two possible explanations, which, however, are not necessarily alternative to each other. The first concerns the concept of the gaze cone (Gamer and & Hecht, 2007), an “area in space that is defined by the range of gaze directions that an observer will accept as directed at him or her” (Horstmann and Linke, 2021, p. 1061). The findings reported by Horstmann and Linke support the idea of the gaze cone subtending a constant visual angle (according to the literature, the range is between ~5 and 10°; Gamer and & Hecht, 2007; Balsdon and & Clifford, 2018; Horstmann and Linke, 2021). The gaze cone metaphor implies that the impression of being looked at should become stronger as the distance between the *looker* and the person who feels looked upon increases. This might account for our findings: a very strong impression that Mona Lisa is looking directly at the observer from more distant observation distances. Nevertheless, Horstmann and Loth (2019) report Mona Lisa’s gaze to be directed 15.4° to the right of the observer, a visual angle which is way beyond the range of the cone of gaze. Hence, if the cone of gaze is responsible for the switch in impression between relatively close and relatively far observation distances, we must assume that, at least in Mona Lisa’s case, the cone is not constant in shape, as the visual angle it subtends would appear to increase with distance.

The second explanation that might account for the differences in findings between Horstmann and Loth (2019) and our findings applies in particular to the results of the nearest observation position (55–110 cm), for which we found that Mona Lisa’s gaze is intrinsically ambiguous. It is most likely that the differences between the two experiments may depend on how gaze direction was measured: Horstmann and Loth employed an indirect metric measurement, which may or may not coincide with one’s impression, whilst we relied only on impressions by asking directly whether the portrait was staring or not at the participant.

Results from experiment 2 for the participants who evaluated Mona Lisa’s gaze seeing the whole head support the results from exp. 1, showing that with relatively big retinal images of Mona Lisa’s head, observers tend to divide into two groups, those who perceive her gaze on them and those who do not. However, results from the group that only saw a section with the eyes of Mona Lisa clearly support the findings reported by Horstmann and Loth (2019). The discrepancy between the data for whole-head vs. eyes-only suggest an important role of Mona Lisa’s head posture in the statistical ambiguity we found with respect to the direction of her gaze with relatively big retinal images.

Results from experiment 1 also show that the impression created by Mona Lisa’s gaze is dynamic, as it is likely to change more towards the impression that she is not staring at the observer as the observer moves closer to the image instead of moving further away (which, on the other hand, supports the hypothesis that the visual angle subtended by Mona Lisa’s cone of gaze increases with distance).

In conclusion, regardless of any measurement, one might want to perform on the direction of Mona Lisa’s gaze, when conducting research in visual perception the priority is to study what is *actually* perceived, not what one ought to perceive based on the geometry of a scene or the theory ones adheres to (Actis-Grosso and Zavagno, 2015). Hence, the fact remains that from afar (or with relatively small retinal images) the perception of Mona Lisa staring at the observer is prominent; from up close, or with relatively big retinal images, the impression becomes statistically ambiguous.

These findings are quite interesting, as they defy the very concept of image robustness: the painting’s scenographic background, Mona Lisa’s torso, and hands do not change much when one moves horizontally, or back and forth, while looking at the image; however, depending on the distance from the portrait and whether one moves closer or further away, the direction of her gaze may shift from staring at the observer to overlooking her/his left shoulder. What is the relationship between a portrait that always follows you with its eyes and the robustness of other surfaces in the portrait that do not seem to change as one moves about?

### On the “Robustness” of Images

The impression of the eyes following is a personal one, and if two people are staring at the same portrait from different positions, both will claim that the portrait is staring at them, which would be impossible if the portrait were instead a real person. Hence, the so-called “Mona Lisa effect” is best described as one of “omnidirectional staring”. The illusion is obviously not limited to Mona Lisa, and it is certainly not limited to gazes: a very similar illusion can be found, for instance, in the posters designed by Alfred Leete in 1914 and by James Montgomery Flagg in 1917, depicting respectively Lord Kitchner and Uncle Sam staring and pointing their index fingers at the observer. Both gazes and fingers continue to follow and to point at viewers as they move about while looking at those images. Given that the illusion does not concern Mona Lisa alone, and that it is not limited to gazes, we dubbed the illusion as the *omnidirectional pointing phenomenon* (OPP).

For decades both common observers and visual scientists have been intrigued by OPP. However, if one considers OPP with respect to picture perception in general, a bit of wonder washes away. We believe, in fact, that OPP falls within the more general phenomenon known as *robustness of perspective* (Kubovy, 1986), a phenomenon that in reality extends to all images, not only to those created with linear perspective. This phenomenon consists in the fact that pictures created by obeying the rules of linear perspective do not appear distorted when not viewed from the correct position (i.e., standing right in front of the vanishing point) despite projections on the retina of such representations may undergo dramatic changes as the observer moves in space while looking at the pictures. According to recent literature, the robustness of perspective does not appear to be “all or none”: structures in such pictures show some degree of distortion when viewed from different angles (Todorovic, 2008), strongly suggesting a graded phenomenon (Pagel, 2017). Nevertheless, the phenomenological experience one has is that a picture’s layout remains the same; that is, distortions are not consciously experienced or, maybe, they are just overlooked by the observer (probably because of the double nature of picture perception; Gibson, 1979; Hagen, 1986; Zavagno, 2007; Pagel, 2017), unless one tries to measure them. In this sense, the experience of robustness is most likely common to all pictures regardless of the geometry adopted to create them. To understand this point, we must consider what the robustness experience is all about, from a phenomenological point of view. Let us first consider the case of pictures that obey the rules of linear perspective: moving in front of one of such pictures determines a series of retinal projections that all differ from one another to some degree; the impression, however, is that the pictorial scene does not change in relation to a rather large range of viewing angles not orthogonal to the vanishing point. In fact, to perceive major distortions the eccentricity of one’s position with respect to the vanishing point must be dearly great. This, of course, unless the image is made such as to appear as an extension of the real world. Such works are known as *trompe-l’oeil*, i.e., optical illusions—see for example the case of Andrea del Pozzo’s (1642–1709) ceiling in the Church of St Ignatius in Rome (Pirenne, 1970; Kubovy, 1986).

Now, if we consider a figurative picture not created according to the set of rules of linear perspective, as for example a child’s drawing, or even something much more sophisticated such as the *Madonna in the Church* by Jan van Eyck (1325–1441; Figure 4) which displays a rather intuitive sense of spatial perspective, we can move in front of the image and the geometry of the church, along with the Madonna with child, will appear more or less unchanged.

**Figure 5:**
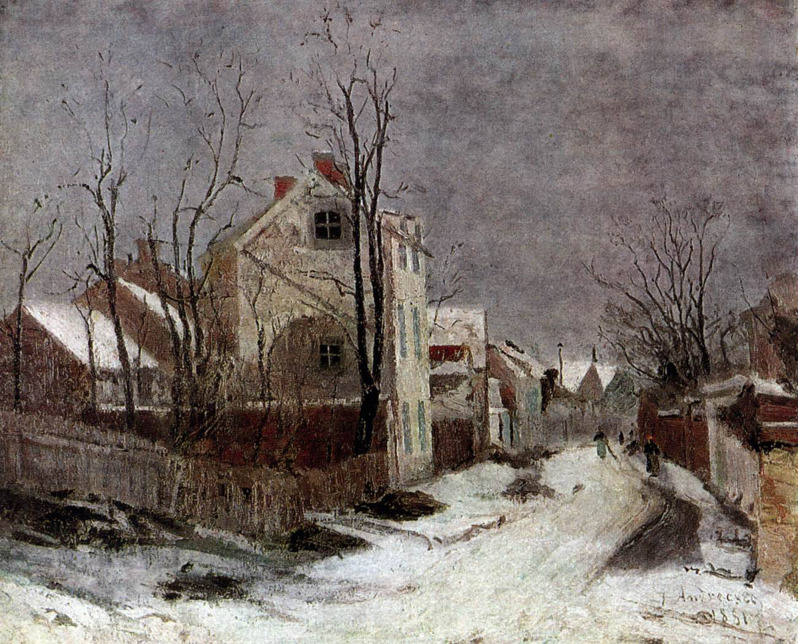
Ion Andreescu, *Winter at Barbizon* (1881, Muzeul K.H. Zambaccian, Bucharest).

What does all this have to do with OPP? Let us consider the painting *Winter at Barbizon* in Figure 5: in this marvelous wintery painting by the Romanian artist Ion Andreescu (1850–1882) we can see a road that from afar proceeds frontally towards us, to then turn slightly to our left. If one moves sideways with respect to the image, one should notice that the portion of the road that appears more in distance (where the people are), right before the curve, is always facing us (always pointing in our direction), while the portion of the road that appears closer, right after the curve, never seems to really point at us. This is one of the basic facts pertaining to picture robustness: if something appears frontal to the observer it always will; if something does not, it never will.

**Figure 6:**
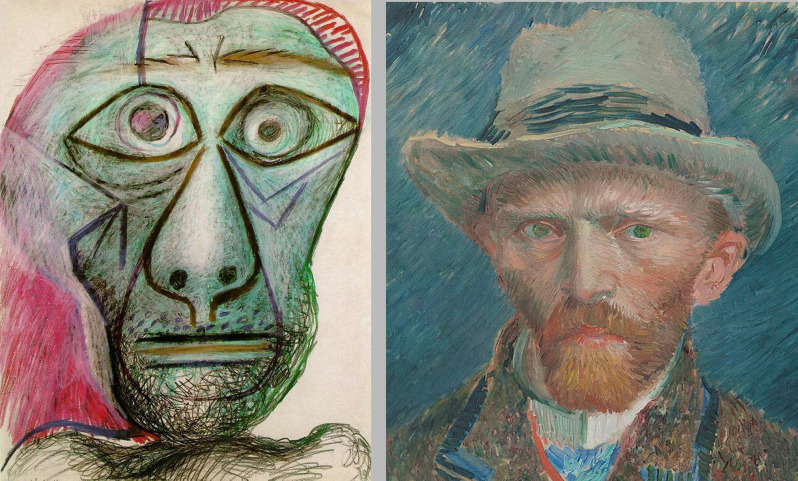
On the left, Pablo Picasso, *Self-portrait facing death* (1972, Fuji Television Gallery, Tokyo). On the right, Vincent van Gogh, *Self-portrait* (1887, Rijksmuseum, Amsterdam).

Now let us consider the two images in Figure 6 on the left, one of Pablo Picasso’s (1881–1973) last self-portraits; on the right, a self-portrait by Vincent Van Gogh (1853–1890). While Picasso’s self-portrait is starring in our direction, Van Gogh’s is looking slightly to our right. We can attempt to move sideways to avoid Picasso’s troubled stare; or move to our right to capture van Gogh’s serious gaze, but all our attempts are doomed to fail. Picasso will continue to stare at us, and van Gogh will continue to avoid looking at us, no matter how hard we try. In both cases, the images show a strong resistance to distortion. In other words, they both prove to be *robust* to the point that the perceived directions of the depicted gazes never shift. Each of those gazes is like a portion of the road depicted by Andreescu. In this sense, both OPP and what we here playfully name the *never pointing phenomenon* (NPP) are actually one and the same thing, both being just instances of picture robustness. We believe that once the mechanisms driving picture robustness will be uncovered, then also the mystery behind OPP and NPP will vanish.

Getting back to Mona Lisa, is her portrait robust or is it a rare deviation from picture robustness? This, we believe, is to be the really challenging question.

## Conclusions: Et in Questo Di Lionardo Vi Era Un Ghigno Tanto Piacevole

Mona Lisa has and will continue to cause much ink to flow. We certainly have not unraveled the enigma behind her expression, nor do we think this to be entirely possible, or even necessary. It is clear, though, that her eyes play a big part in our fascination, whether we are aware of this or not. We are not certain whether her smile is uncatchable, as suggested by others (Livingstone, 2000; Soranzo and Newberry, 2015), but we are certain that her gaze is even more ambiguous than her smile, and that it might be truly considered uncatchable, unless looked from afar. In this sense, Da Vinci’s painting defies the typical robustness of pictorial images without being a scenographic *trompe l’oeil*: even most of those who do not see her looking at them when viewing a big, decent reproduction of the painting, will have the impression of her gaze following them when the retinal image is relatively small, as when visiting the Louvre or looking at a reproduction in a journal. When instead the image is big enough, people will divide spontaneously into two groups, those who see her staring at them, and those who see her looking aside.

The short passage that entitles our conclusive paragraph is taken from the description of the portrait of Mona Lisa written by Giorgio Vasari; the passage concludes: *…che era cosa più divina che umana a vederlo, et era tenuta cosa maravigliosa, per non essere il vivo altriment i* (Vasari, 1991). The whole passage has been translated into English as such: “And in this work of Leonardo’s there was a smile so pleasing, that it was a thing more divine than human to behold; and it was held to be something marvellous, since the reality was not more alive” (Vasari, 1914).

When describing the painting, Vasari mentions eyebrows and “lustri” (lights) in the eyes, which, however, are both absent in the painting. Some say due to excessive cleaning, however, without providing proof. Vasari never really saw the original painting, which most likely was sold after Salai’s death (Gian Giacomo Caprotti, 1480–1524, one of Leonardo’s favorite apprentices) to Francis I, along with other paintings by Leonardo. Hence, Vasari’s description is second hand, probably based on descriptions by others and/or copies of the painting (drawings, etchings) that might have been circulating, maybe created by Salai himself or another of Leonardo’s apprentices; after all, that is how ideas circulated in the *artworld* of the time (Danto, 1964)^1^. What is remarkable about Vasari’s description is that word of the effect of Mona Lisa’s expression was already circulating, as testified by Vasari’s use of the terms *ghigno tanto piacevole* to describe Mona Lisa’s facial expression. The word *ghigno* does not simply refer to a smile, rather it refers to a complex facial expression and it has always retained a negative meaning, best translated from Italian with “malevolent smile”, “sneer”. Hence, the official English translation does not do justice to Vasari’s linguistic skills, it does not render the sense of his oxymoron. By accosting the adjective “pleasant” to sneer, Vasari transformed Mona Lisa’s smile into an enigma, best translated not simply with a “pleasant smile”, as reported by the English translation, but with a “rather playful smile”.

Why did Leonardo never depart from his painting? Why didn’t he sell it to Francesco del Giocondo, Mona Lisa’s husband who commissioned the painting (hence the Italian name for the painting, *La Gioconda*)? Of course, we do not have an answer to such questions, and we do not know if the painting is a portrait of Monna Lisa, Francesco del Giocondo’s wife. What we propose from here on is mostly an academic exercise, that, however, might hold a pinch of truth. If not, it is still a rather entertaining hypothesis.

The hypothesis that *La Gioconda* was dear to Leonardo because he realized that he had achieved one of the goals that he set in one of his many annotations, which would become known as *Trattato della pittura*, probably put together by Francesco Melzi (1491–1568), another of Leonardo’s apprentices who inherited many of his manuscripts and drawings. A goal that an artist should set, according to the *Trattato*, is to represent *accidenti mentali* and *moti mentali*, the first to be intended as emotions and affective states, the second as thoughts and motivations. For example, in the annotation 372 entitled *Che se le figure non esprimono la mente sono due volte morte* (Da Vinci, 1804), Leonardo writes: *Se le figure non fanno atti pronti i quali colle membra esprimano il concetto della mente loro, esse figure sono due volte morte, perché morte sono principalmente ché la pittura in sé non è viva, ma esprimitrice di cose vive senza vita, e se non la si aggiunge la vivacità dell’atto, essa rimane morta la seconda volta* (Leonardo, 1804, p. 192; “How figures not expressive of the mind are twice dead. If figures do not make lifelike gestures with their limbs which express what is passing through their minds, these figures are twice dead”. Pedretti, 1964, p. 46).

Portraits do not normally portray actions, and in particular, Mona Lisa’s posture is rather statuesque, showing the final stages of Leonardo’s artistic interests, with the torso blocked in a half twist (Marani, 1994). However, out of will, or by chance, Leonardo infused in Mona Lisa’s facial expression “motion”. Not the motion that characterizes facial expressions such as anger, fear, joy, or sadness, which can induce an implicit sense of dynamism to the figure represented (Actis-Grosso and Zavagno, 2015; Della Torre et al., 2021), but something much more subtle, elusive, like someone whose gaze is lost in the void while looking in the direction of another person. *Is she looking at me?* Leonardo managed to depict an actual *moto mentale*, the act of thinking that often characterizes such stares. As an acute observer of all the phenomena he encountered, Leonardo must have realized right away that the impressions generated by the portrait went far beyond different interpretations of Mona Lisa’s facial expressions by different observers; he might have also noticed that different distances of observation of the painting changed one’s own interpretation of her gaze/expression, resulting in an image that appeared alive. The final words by Vasari, *et era tenuta cosa maravigliosa, per non essere il vivo altrimenti*, describe exactly this.

The *moto mentale* he managed to portray through Mona Lisa may be the reason why he did not insert “highlights” in the eyes, which would have fixed forever Mona Lisa’s gaze in one direction, as shown in a work in preparation in which highlights were placed in Mona Lisa’s eyes in different positions (Succi, Avanzi, and Zavagno, in preparation). This may also be the reason why the eyebrows are missing, as they also would have contributed to fixing her facial expression. Both highlights and eyebrows are instead visible in an interesting copy of Mona Lisa that can been seen in the Prado Museum, datable between 1503–1519 and painted by one of Leonardo’s apprentices. Maybe *La Gioconda* had become a portable experiment for Leonardo, with which he could amuse those who visited him, while studying their reactions.

As said previously, ours is only academic speculation. What matters for a psychology of art is that regardless of the *actual* direction of Mona Lisa’s gaze, this is modulated by distance and all kinds of people, not only scientists, either feel her gaze upon or beyond them, all remaining blissfully puzzled by her playfully enigmatic expression. Could this be the effect that Leonardo was looking for? We do not have an answer to such a question. But we can say one thing: in some sense, the painting *La Gioconda* is more ambiguous than robust, maybe because Leonardo depicted an *affective trompe l’oeil*.

## Data Availability Statement

The raw data supporting the conclusions of this article will be made available by the authors, without undue reservation. Link to data is this: https://www.researchgate.net/publication/361053337_Data_exp1_MonaLisaFrontiers.

## Ethics Statement

Ethical review and approval was not required for the study on human participants in accordance with the local legislation and institutional requirements. The patients/participants provided their written informed consent to participate in this study.

## Author Contributions

DZ was responsible for preparing the manuscript, experimental designs, and data management and analyses. RA-G was responsible for discussing concepts, reviewing the manuscript, and coordinating experiment 1. OD was responsible for discussing concepts, reviewing the manuscript, and coordinating experiment 2.

## Conflict of Interest

The authors declare that the research was conducted in the absence of any commercial or financial relationships that could be construed as a potential conflict of interest.

## Publisher’s Note

All claims expressed in this article are solely those of the authors and do not necessarily represent those of their affiliated organizations, or those of the publisher, the editors and the reviewers. Any product that may be evaluated in this article, or claim that may be made by its manufacturer, is not guaranteed or endorsed by the publisher.

## Footnotes

^1^Artworld is a concept developed by Arthur Danto (20) to explain contemporary aesthetics. The concept, however, with some re-interpretation, can be employed and extended to talk about the production and appreciation of art also in times that are far from our own.
